# A tale of swinger insects: Signatures of past sexuality between divergent lineages of a parthenogenetic weevil revealed by ribosomal intraindividual variation

**DOI:** 10.1371/journal.pone.0195551

**Published:** 2018-05-02

**Authors:** Marcela S. Rodriguero, Sonia A. Wirth, Josefina S. Alberghina, Analía A. Lanteri, Viviana A. Confalonieri

**Affiliations:** 1 Departamento de Ecología, Genética y Evolución, Facultad de Ciencias Exactas y Naturales, Universidad de Buenos Aires—IEGEBA (CONICET-UBA), Buenos Aires, Argentina; 2 Laboratorio de Agrobiotecnología, Departamento de Fisiología, Biología Molecular y Celular, Facultad de Ciencias Exactas y Naturales, Universidad de Buenos Aires e Instituto de Biodiversidad y Biología Experimental y Aplicada (IBBEA—CONICET/UBA), Ciudad Autónoma de Buenos Aires, Buenos Aires, Argentina; 3 División Entomología, Museo de La Plata, Facultad de Ciencias Naturales y Museo, Universidad Nacional de La Plata—CONICET, La Plata, Buenos Aires, Argentina; University of Innsbruck, AUSTRIA

## Abstract

*Naupactus cervinus* (Boheman) (Curculionidae, Naupactini) is a parthenogenetic weevil native to the Paranaense Forest which displays high levels of genetic variation. Two divergent clades were identified, one ranging in forest areas (Forest clade), and the other in open vegetation areas (Grassland clade). Both of them have individuals with high levels of heterozygosity in ribosomal sequences. Investigation of intraindividual variation in *ITS1* sequences through cloning and posterior sequencing suggested that mating between both groups most likely occurred in the Paranaense Forest after a secondary contact, which led to fixed heterozygotes as a consequence of parthenogenesis. Otherwise, sexual segregation would have disrupted multilocus genotypes. Only a small number of heterozygous genotypes of all the possible combinations are found in nature. We propose the occurrence of a hybrid zone in the Paranaense Forest. The fact that it is one of the most important biodiversity hotspots of the world, together with its key role for investigating evolutionary processes, makes it worthy of conservation. This is the first genetic evidence of bisexuality in *N*. *cervinus*.

## Introduction

Weevils are unique for evolutionary entomologists because they show many interesting features such as parthenogenesis and polyploidy [1]. In the Old World the genus *Otiorhynchus* (Entiminae, Otiorrhynchini) comprises more than 60 unisexual species distributed in Central and Northern Europe (e.g., [1–5]), where parthenogenetic species of the genus *Polydrosus* are also found [6–7]. Several species of the genus *Cathormiocerus* inhabit the British and Azores Isles [8–9]. In Japan, about 10% of weevils are unisexual [10], and similar numbers were reported for Canada [11]. These species are characterized by a high frequency of polyploidy, especially triploidy [12–13]. Polyploidy in Curculionidae has been found to be associated with the occurrence of parthenogenesis [1,12]. So far, 48 triploid, 18 tetraploid, six pentaploid, three hexaploid and one decaploid lineages have been reported [14], with broad-nose weevils having a modal chromosome number of 11 [2,12]. Parthenogenetic weevils have been more thoroughly studied in the Northern Hemisphere than in Southern Hemisphere. Although this reproductive mode has been proposed for some species belonging to the Neotropical tribe Naupactini [15], it was only demonstrated in a few of them [16], suggesting an underestimation of the true number of parthenogenetic weevils.

The “Fuller’s rose weevil” *Naupactus cervinus* (Boheman) (Curculionidae, Naupactini) is an important pest of orange trees and ornamental plants, mainly in areas of introduction [17–18]. It appears as a promising prospect to gain knowledge about the evolution of parthenogenesis in the South American entomofauna because of its worldwide distribution and abundance. Currently, this species is parthenogenetic over its entire range, as supported by the rearing experiments and genitalia dissection of hundreds of individuals performed by Floyd F. Smith and reported by [16]. However, some sexual lineages were collected in forest areas of northeastern Argentina (Misiones province), and southern Brazil (Santa Catarina State) and some individuals were seen copulating in Brazil in the 1950’s [19]. It is possible that these bisexual populations would have become extinct [20–21].

In the taxonomic study of *Asynonychus* Crotch (current synonym of *Naupactus* Dejean), two species with different morphometric characters were recognized by [22–23], the cosmopolitan parthenogenetic *Asynonychus godmanni* and the subtropical bisexual *Asynonychus cervinus* (Boheman) (described based on material from USA and Brazil, respectively). Subsequently, [24] proposed to unite them into a single species (*A*. *cervinus*, by priority) with geographic parthenogenesis [15,24], defined as the spatial bias in the distribution of taxa with different reproductive mode [25]. Parthenogenesis seems to be strongly related to the great colonisation ability of *N*. *cervinus* [21].

More recently, molecular studies have shed light on the natural history of *N*. *cervinus*. Mito-nuclear coevolution suggests that parthenogenesis is an ancient phenomenon, as indicated by the co-segregation of one mitochondrial haplotype and its derivatives with a given *ITS1* allele [20]. In addition, infection by the sex-ratio distorter bacterium *Wolbachia pipientis* appears as a possible explanation for the origin of this reproductive mode [26–27], although the origin of parthenogenesis in Naupactini weevils has not yet been conclusively elucidated.

*Naupactus cervinus* is a species complex, which encompasses at least two independently evolving and morphologically similar lineages, namely “I” and “II”. Lineage I groups the divergent clades Forest and Grassland [21, 26, 28], with parapatric distribution. Lineage II is mainly found in forests [28]. Both lineages are infected with *Wolbachia* [28].

*Naupactus cervinus* is native to the Paranaense Forest (a humid subtropical forest in southeastern Brazil, eastern Paraguay and northeastern Argentina) and expanded its range southwards during the Pleistocene through the gallery forests along the Paraná and Uruguay rivers, which acted as natural corridors [21]. Further isolation between populations in the current territories of Brazil and Argentina led to their divergence, giving rise to the previously mentioned Forest and Grassland clades of Lineage I (in the north and south of the species’ distribution, respectively). The current distribution of the genetic variants of *N*. *cervinus* suggests that both clades would have come into secondary contact when environmental conditions along the gallery forests became favorable in the Paranaense Forest [21]; then, in the last 100 kyr some Grassland variants would have colonized open vegetation areas (i.e., grasslands and steppes of the Pampa province), which are now highly disturbed by agricultural activities. Parthenogens seem to have been deeply involved in the species’ range expansion during the late Pleistocene-early Holocene, but the role of bisexual populations in this process remains a conundrum.

Notwithstanding its reproductive mode, *N*. *cervinus* depicts high levels of genetic variation as a consequence of the complex demographic processes described above [21, 28]. While surveying for genetic variation over a large portion of the Fuller’s rose weevil distribution range we amplified two different alleles per individual in four Argentinean and nine Brazilian locations of a total of 55 sampled by [20–21, 28]. Thus, we found intra-individual genetic variation in the ribosomal sequence *ITS1* (Internal Transcribed Spacer 1), and called them “peaks on peaks” (hereafter “POP”) because of the superimposed peaks on the electropherogram. Strikingly, all POP sequences were associated with a few mitochondrial haplotypes from both Grassland and Forest clades [20–21]. This exciting finding led us to hypothesize whether an outcrossing event could have occurred between the Grassland and Forest lineages of *N*. *cervinus* as a by-product of a secondary contact during the late Pleistocene-early Holocene. However, mutation in paralogous sequences and incomplete concerted evolution [29], cannot be discarded because ribosomal sequences are tandemly repeated in the genome. Under the first scenario, it is expected to obtain one allele from the Forest clade and another one from the Grassland clade. Alternatively, we could find closely related alleles with at least one insertion/deletion event between paralogous copies.

Our results can be explained by the occurrence of outcrossing between divergent lineages or mutations in paralogous copies of *ITS1* coupled with incomplete concerted evolution. The latter scenario is less likely to occur because mutation in paralogous sequences would yield a random pattern of allelic variation.

Our paper aims to gain further insight on the demographic history, mode of reproduction and concerted evolution of multigene copies in *N*. *cervinus* in an attempt to shed light on the evolution of parthenogenesis in Naupactini weevils. For this purpose, we analyzed intra-individual *ITS1* variation in the samples of *N*. *cervinus* containing the individuals with POP *ITS1* mentioned above.

## Materials and methods

### Specimens examined

**Fig 1:**
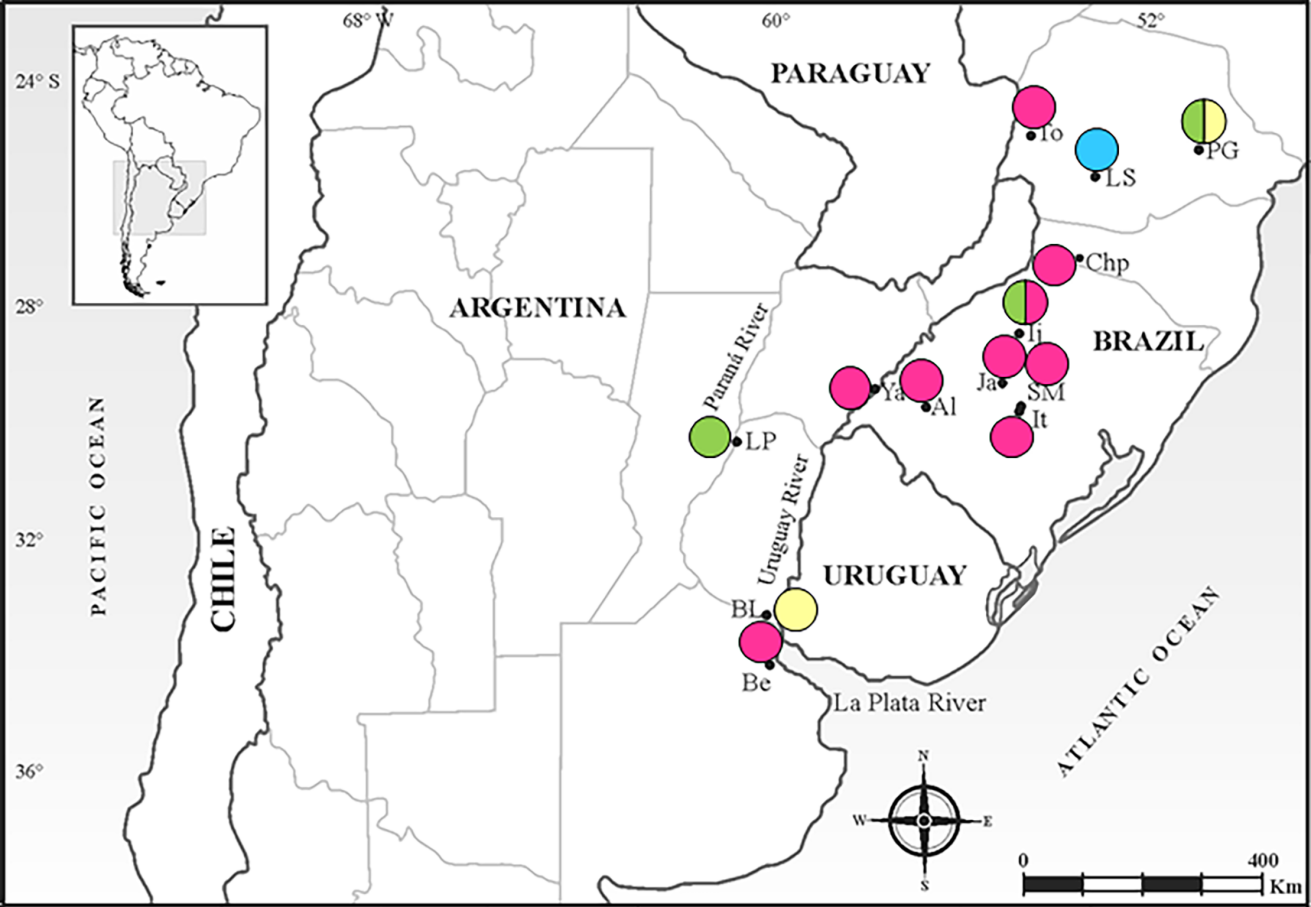
Map showing the sampled localities of *Naupactus cervinus* and the genetic constitution of heterozygous genotypes. For acronyms of localities see Table 1. Green I—XIX genotypes; blue: XIII—XII genotypes; yellow: VI—XVIII genotypes; pink: VI—VIII genotypes; green/pink: coexistence of I—XIX and VI—VIII genotypes; yellow/green: coexistence of I—XIX and VI—XVIII genotypes.

Previous surveys of *N*. *cervinus* conducted in wild and cultivated plants from several geographic locations showed that 84 of 400 (21%) individuals from nine Brazilian and four Argentinean locations carried POP *ITS1* sequences [20–21]. In this work, our analysis will be focused on all the 149 individuals collected from these 13 sampling points (Table 1; Fig 1).

**Table 1:** Frequency of heterozygotes.

Location	Acronym	Latitude/Longitude	N	Genotype	Frequency
Alegrete (BR)	Al	29° 46’ S, 55° 47’ W	7	C **H**	0.150
Benavídez (AR)	Be	34° 24’ S, 58° 41’ W	7	C **H**	0.850
Brazo Largo (AR)	BL	33° 54’ S, 58° 53’ W	15	C **H**	0.250
Chapecó (BR)	Chp	27° 03’ S, 52° 36’ W	8	E **H**	0.125
Ijuí (BR)	Ij	28° 23’ S, 53° 54’ W	8	C **H**	0.500
				R **H**	0.250
Itaara (BR)	It	29° 36’ S, 53° 45’ W	10	C **H**	0.700
Jari (BR)	Ja	29° 17’ S, 54° 13’ W	17	C **H**	0.700
La Paz (AR)	LP	30° 45’ S, 59° 38’ W	6	R **H**	1.000
Laranjeiras do Sul (BR)	LS	25° 24’ S, 52° 24’ W	13	V **H**	0.770
Ponta Grossa (BR)	PG	25° 05’ S, 50° 09’ W	14	R **H**	0.290
				C **H**	0.360
Santa Maria (BR)	SM	29° 40’ S, 53° 47’ W	20	C **H**	0.150
Toledo (BR)	To	24° 42’ S, 53° 44’ W	19	C **H**	0.700
Yapeyú (AR)	Ya	29° 28’ S, 56° 50’ W	5	C **H**	0.800
				E **H**	0.200

Georeferenced locations considered in this study with the frequency of the heterozygous multilocus genotypes (*COI*-*ITS1*) ranging in the area. C, E, R, V = mitochondrial alleles [21]; H (in bold) is for “heterozygous genotype” (POP *ITS1* individuals). N: total number of individuals collected (with homozygous and heterozygous genotypes). BR: Brazil; AR: Argentina.

After visual comparison of *ITS1* chromatograms of these individuals, we recovered four groups of POP sequences, each of which is associated with one of the four mitochondrial haplotypes R, V, C, and E (a derivative from C, see below). To study intraindividual *ITS1* allelic variation, in this work we randomly selected one to five individuals per geographic location of every group (N = 27). We also included *ITS1* sequences obtained from *Naupactus dissimulator* and *Pantomorus postfasciatus* as outgroup (GenBank Accession Numbers GQ406826.1, JX440501.1- JX4405035.1, JX440498.1).

### PCR assay, cloning and sequencing

A region of about 1100 bp was amplified using the primers rDNA2 and rDNA 1.58S, which are suitable for amplifying the region 3’ of the *18S rDNA* gene, plus the complete *ITS1* region and the 5’ region of the *5*.*8S rDNA* gene [20–21, 28].

Amplification was carried out in a 50 μl volume reaction with 50–100 ng of DNA used as template, 0.5 μM of each primer (Thermo Scientific, Rockford, IL, USA), 0.1 mM of each dNTP (Promega, Madison, WI, USA), 3.0 mM MgCl_2_, 1 unit of Taq polymerase and 1X reaction buffer (Thermo Scientific, Rockford, IL, USA). The reactions were performed in a GeneAmp® PCR System 2700 thermal cycler (Applied Biosystems, Inc., Gaithersburg, MD, USA) under the conditions described by [20–21, 28].

Double—stranded PCR products were separated by electrophoresis on a 1% agarose gel with TAE buffer containing GelRed^TM^ (GenBiotech, Buenos Aires, Argentina). The PCR products were purified with a QIAquick Gel Extraction Kit (Qiagen Inc., Valencia, CA, USA).

The PCR products of *N*. *cervinus* were cloned into the pGEM-T easy-cloning vector (Promega, Madison, WI, USA) in order to separate ambiguous sites between the different intraindividual *ITS1* copies. Four to twelve clones per each amplification product were isolated, and *ITS1* inserts were sequenced using the vector primers T7 and SP6. DNA was sequenced using a 3130-XL Automatic Sequencer (Applied Biosystems, Inc., Foster City, CA, USA).

Standard PCR of multiallelic loci has the potential to create chimeric products when single strands of unfinished PCR products of one allele prime the reaction in a subsequent cycle using a strand of the other allele. These chimeras are often hidden or appear as slightly "dirty" sequences when PCR products are sequenced directly. However, if PCR products are cloned, they may be isolated and taken as allelic variants. To avoid this bias, we sequenced many clones from every individual assayed. We also eliminated sequences with singletons that were present neither in any other clones from the same individual nor in the alleles already reported for *N*. *cervinus*.

Given that *Wolbachia* might have a role in the onset of parthenogenesis, among other factors, in Naupactini weevils [26–27], it is possible that this endosymbiont might have been involved in the interclade mating discovered in the present report. Identification of the strains in all the populations included in the present work might help to approach this hypothesis.

For *Wolbachia* variation analysis, we sequenced the two most variable genes of the MLST system in Naupactini, *fbpA* and *hcpA*, in one individual from each location and genotype [26]. We used the primers and conditions described by [30]. Identical nucleotide sequences on a given locus were assigned to the same arbitrary allele number after comparison with the *Wolbachia* MLST database (http://pubmlst.org/wolbachia). Then, each strain was characterized by the combination of the fbpA and hcpA numbers. Strain information was compared with the MSLT of strain *w*Nau5 described by [26].

### Data analysis

#### Alignment

Electropherograms of forward and reverse sequences were edited using the program Bioedit v7.0.5 [31]. Alignment of cloned sequences with respect to the other alleles already identified for the *ITS* gene fragment from the Grassland (V, VII-XII, XV-XVIII) and Forest (I-IV, VI, XIII, XIV) lineages [21,26,28]) was done using CLUSTAL W v2.1 [32]. The mitochondrial locus was previously characterized by [20–21, 28] for both Grassland (A-N, V-X) and Forest (O-R) clades (see Table 1).

Because *ITS1* sequences typically evolve by sequence slippage in a single evolutionary event [33–34], in this analysis we used two types of gap-coding strategies: 1.- each gap is considered as an independent gain/loss event (equal gap-opening and gap-extension penalties), and 2.- gap-opening cost is assumed to be much higher than gap-extension cost (and thus only the opening gap is considered as a single evolutionary event). Both alignment methods yielded the same result (data not shown).

#### Phylogenetic analysis

To disentangle the relationships between the clones isolated in the present work, we performed a phylogenetic analysis of *ITS1* alleles using alleles as “terminal taxa”. We used the program MrBayes v3.2.3 [35]. The optimal model of nucleotide substitution was HKY + G, which was estimated with jModelTest v2.1.7 [36–37] using Phylemon v2.0 [38] on the basis of the Bayesian Information Criterion (BIC). Bayesian analysis was performed implementing the ‘Metropolis-coupled Markov chain Monte Carlo’ (MCMCMC) algorithm. Two independent analyses using four chains, one cold and three incrementally heated, were run using a random starting tree over 2,000,000 generations sampling every 500 generations. The average standard deviation of split frequencies stabilized to a difference of < 1%, and the software Tracer v1.6 [39] was used to assess convergence of the cold chain. The initial 250,000 generations from each run were discarded as burn-in. Tree parameters and topology were visualized with FigTree v1.4.3 [40].

### Divergence time estimation

The estimation of the divergence time between the closely related mitochondrial haplotypes C and E could be used to determine a time frame to the occurrence of one of the outcrossing events in *N*. *cervinus*.

We used a strict-clock analysis with a coalescent prior as implemented in BEAST v1.8.4 for reconstructing an ultrametric tree [41]. The DNA substitution model was chosen as explained above (GTR + I + G). We run one MCMC chain for 50 million generations and sampled every 1,000 generations, resulting in 50,000 trees for each run. The first 5,000 trees were then discarded. The maximum clade credibility tree found using TreeAnnotator v1.6.2 [41] with all options set to default was used as input data to recover the (C-E) divergence time.

We chose a nucleotide substitution rate of 0.0177 substitutions/site/Myr [42]. Relative-rate tests were performed to test the equality of evolutionary rates between lineages [43] using MEGA v6.0 [44].

## Results

### Variation in *ITS1* sequences and phylogenetic reconstruction

Overall, POP individuals were 56% of the sample (84 out of 149 individuals, Table 1). POP genotypes were present at high frequencies in some locations (e.g. Benavídez, Ijuí, La Paz, Yapeyú) and at low frequencies in others (e.g. Alegrete, Brazo Largo, Santa Maria) (Table 1). Seventy two percent of heterozygotes were associated with the Grassland mitochondrial haplotype C, 15% with the Forest mitochondrial haplotype R, 11% with the Forest mitochondrial haplotype V and 2% with the Grassland mitochondrial haplotype E.

We obtained *ITS1* sequences about 1100 bp long for 127 clones from 27 individuals of *N*. *cervinus* with heterozygous genotype. All of them were females, as observed in previous works [20–21]. Multiple alignment of the whole set of *ITS1* alleles known for *N*. *cervinus* with those obtained in the present work (i.e. 127 clones) and other *ITS1* sequences from other *Naupactus* species yielded a matrix of 961 bp in length and showed 97 substitutions and ten deletions/insertions (between 1–9 bp in length). This alignment revealed that heterozygous individuals carried alleles already reported for *N*. *cervinus* [21, 26, 28], namely I, VI, VIII, XII, XIII and XVIII (GenBank Accession Numbers GQ406818.1, GQ406824.1, GQ406825.1, KX074094.1, KX074088.1, KX074089.1, respectively) (Table 2). In the present paper we found a new allele: XIX (GenBank Accession Number KY305942.1).

**Table 2:** Genetic constitution of heterozygotes.

Location	Acronym	N	Nuclear Genotype	Mitochondrial Haplotype
Alegrete	Al	1	**VI—**VIII	C
Benavídez	Be	1	**VI—**VIII	C
Brazo Largo	BL	4	**VI—**XVIII	C
Chapecó	Chp	1	**VI—**VIII	E
Ijuí	Ij	1	**VI—**VIII	C
		1	**I—**XIX	R
Itaara	It	1	**VI—**VIII	C
Jari	Ja	1	**VI—**VIII	C
La Paz	LP	1	**I—**XIX	R
Laranjeiras do Sul	LS	3	**XIII—**XII	V
Ponta Grossa	PG	1	**VI—**XVIII	C
		1	**I—**XIX	R
Santa Maria	SM	1	**VI—**VIII	C
Toledo	To	5	**VI—**VIII	C
Yapeyú	Ya	2	**VI—**VIII	C
		1	**VI—**VIII	E

Forest alleles are in bold and Grassland alleles in regular type. N = number of individuals used in the cloning experiments.

**Fig 2:**
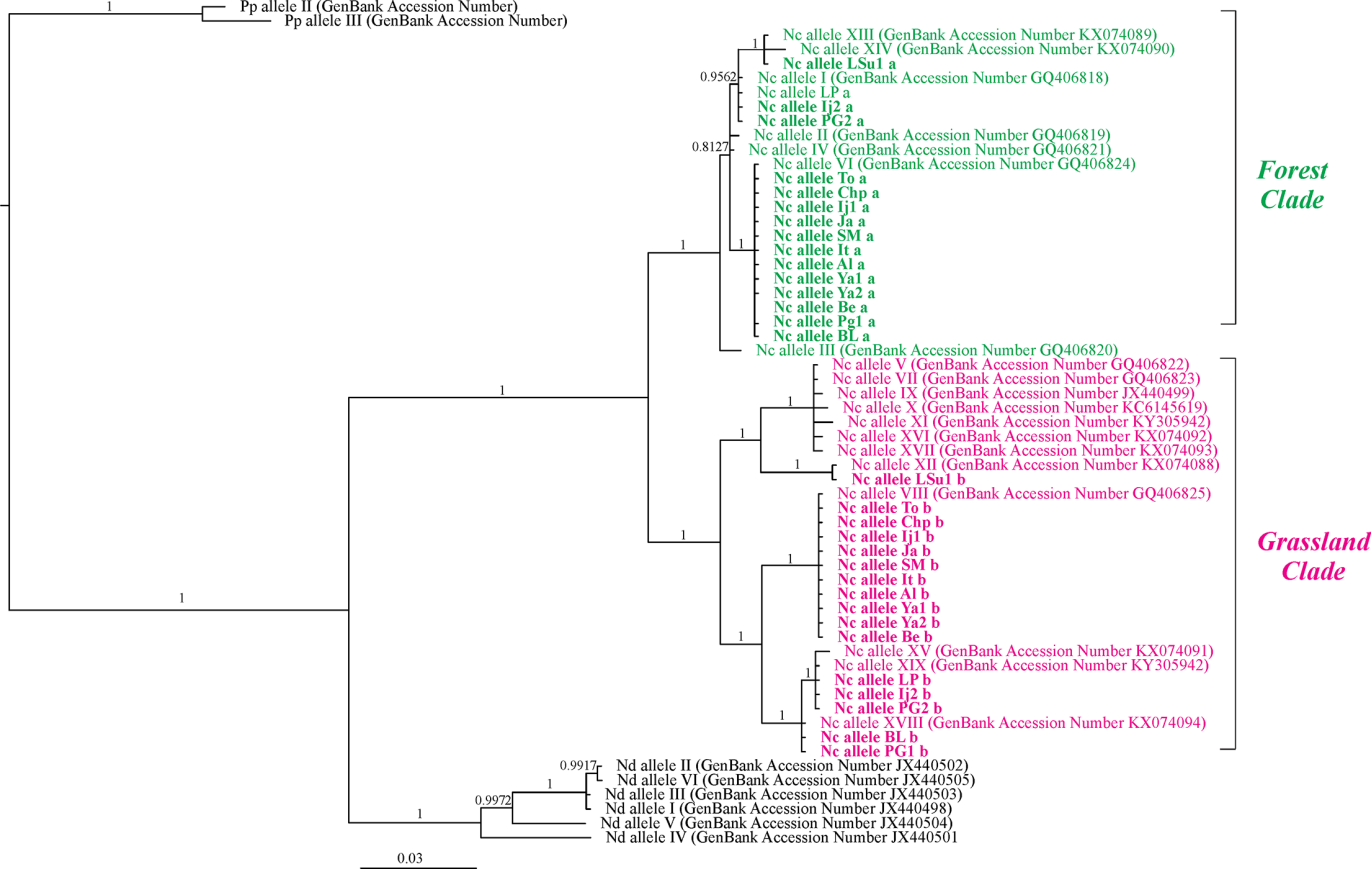
Phylogram of *Naupactus cervinus ITS1* alleles. Alleles from heterozygous individuals are labelled with the acronym of the location, an individual ID, and a clade ID (a: Forest, b: Grassland). Green: Forest clade alleles; Pink: Grassland clade alleles. Copies obtained in the present analysis are in bold. Numbers above the branches are 50% or higher posterior probability values. Nc = *Naupactus cervinus*; Nd = *Naupactus dissimulator*; Pp = *Pantomorus postfasciatus*.

Bayesian inference based on this matrix recovered both Forest and Grassland clades as in previous works [20–21, 28] (Fig 2). This analysis also confirmed the identification of the alleles revealed by multiple alignment, because sequences obtained from heterozygous individuals in this work (indicated with the population acronym plus a letter "a" or "b" corresponding to both alleles found in each genotype) were clustered together with their corresponding allele already identified in previous analyses. The new allele XIX grouped with the Grassland alleles XV and XVIII (Fig 2). In the Bayesian tree, the branches of the Grassland clade are much longer than those of the Forest clade.

The analysis of intra-individual variability revealed that one of the two *ITS1* alleles for each genotype belongs to the Forest clade whereas the other belongs to the Grassland clade (Fig 2). These results would indicate the occurrence of outcrossing between *N*. *cervinus* males and females from the Forest and Grassland clades.

Individuals from Alegrete, Benavídez, Chapecó, Ijuí, Itaara, Jari, Santa Maria, Toledo and Yapeyú carrying the mitochondrial haplotypes C and E (Grassland clade) have the *ITS1* allelic combination VI-VIII (64% of the POP individuals, Table 1, Fig 1). Weevils from Brazo Largo and Ponta Grossa, which also show the mitochondrial *COI* haplotype C, share the combination VI-XVIII (11% of the POP individuals, Table 1, Fig 1). On the other hand, specimens from Ijuí, La Paz and Ponta Grossa harbor the *ITS1* allelic combination I-XIX in association with the mitochondrial *COI* haplotype R (Forest clade) [20–21] (Table 1; Fig 1). For Laranjeiras do Sul we obtained another combination, XIII-XII, in individuals exhibiting the mitochondrial haplotype V as previously reported by [21] (Forest clade) (Table 1; Fig 1. No variation was found within locations excepting for Ijuí and Ponta Grossa (Table 2; Fig 1).

The *ITS1* allele VIII is only associated to the mitochondrial haplotype C [20], and this genotype appears in the Brazilian locations of Alegrete and Itaára, while the allele I was always found associated with the mitochondrial haplotype R and it occurs in the Brazilian location of Ponta Grossa. Except for POP individuals, no association was found between the mitochondrial haplotype C and the genotype VI in any location across 400 sampled individuals [21].

### Minimum age of the C (VI-VIII) outcrossing event

Time frame preceding outcrossing could be estimated on the basis of the number of nucleotide substitutions accumulated from mitochondrial haplotype C to mitochondrial haplotype E (Genbank Accession Number GQ406829.1 and GQ406831.1, respectively), based on the assumption that the E (VI-VIII) lineage derived from the C (VI-VIII) lineage [21]. Therefore, the diversification of the C into the E haplotype would have occurred after outcrossing between the former and some other genotypes. This assumption is based on the following observations: i) the mitochondrial network in [21]; ii) E was not found in combination with any homozygous *ITS1* genotype in the whole sample studied by [20–21, 28]; iii) E was always found in POP individuals associated with VI-VIII. The divergence time for this haplotype pair would be approximately 190 kya (95% HPD: 0 to 290 kya).

### *Wolbachia* variation

*Wolbachia*’s alleles infecting the heterozygous individuals coincided with those of the *w*Nau5 strain already reported for *N*. *cervinus* [26]: the *fbpA -*allele 9 and the *hcpA—*allele 126. In short, all the genotypes studied were infected with the same strain of *Wolbachia*, and the same already reported for all the homozygous genotypes of *N*. *cervinus* [20].

## Discussion

Intraindividual variation in the sequence of ribosomal genes is unusual in animal genomes, although it has been reported for some fishes and corals in relation to polyploidy, hybridization and a reduced rate of concerted evolution [6–7, 45–46]. In this work, we found heterozygous genotypes for *ITS1* sequences in the parthenogenetic weevil *N*. *cervinus*. Our results can be explained by the occurrence of outcrossing between divergent lineages or mutations in paralogous copies of *ITS1* coupled with incomplete concerted evolution. The latter scenario is less likely because mutation in paralogous sequences would yield a random pattern of allelic variation. Instead, both copies recovered from each POP individual have already been seen in homozygous genotypes of *N*. *cervinus*. Thus, the former scenario is more plausible.

Based on the linkage disequilibrium between mitochondrial and nuclear variants C-VIII and R-I [20], we infer that alleles VIII and I might have resulted from maternal contribution of C (VI-VIII) and R (I-XIX) genotypes, respectively. Therefore, the nuclear alleles VI and XIX might have originated from paternal contribution. These assumptions are supported by the finding of C-VIII and R-I females in the proposed area of secondary contact, as stated in [21].

**Fig 3:**
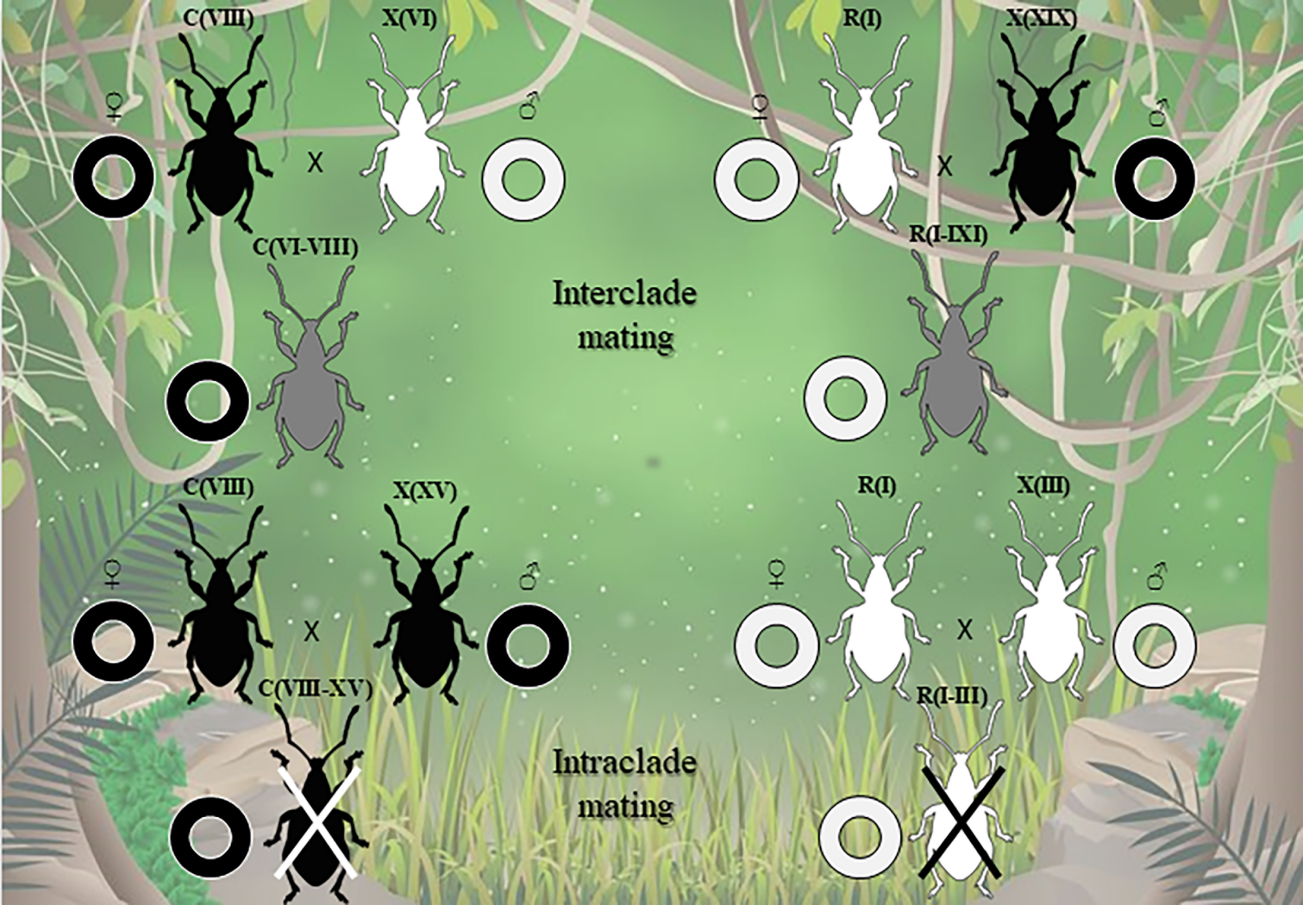
Schematic representation of a possible scenario illustrating interclade and intraclade matings between *Naupactus cervinus* individuals in the Paranaense Forest. F: Forest clade, black weevils; G: Grassland clade, white weevils. Rings represent mitochondrial lineages (black: Forest clade; white: Grassland clade). Under this scenario, only a few interclade matings (i.e. ♀ C VIII x ♂ VI; ♀ R I x ♂ XIX, etc.) -among all possible combinations- would have yielded viable progeny (gray weevils), while no intraclade mating would have ever produced viable progeny.

According to [20–21], who reported that C and E are Grassland mitochondrial haplotypes, females carrying heterozygous genotypes C (VI-VIII), C (VI-XVIII) and E (VI-VIII) would be descendants of a mother from the Grassland clade and a father from the Forest clade. Likewise, those bearing the R (I-XIX) and V (XIII-XII) genotypes would derive from a mother of the Forest clade and a father from the Grassland clade, since R and V are mitochondrial haplotypes belonging to the Forest clade [20–21]. We propose that interclade crosses were produced in the forest, which can be considered as an area of secondary contact between divergent lineages. Consequently, these results provide the first genetic evidence of bisexuality in *N*. *cervinus*. The origin of heterozygous genotypes from *ITS1* sequences is diagrammed in Fig 3.

Considering that the VIII and XVIII variants are not closely related (Fig 2), the genotypes C (VI-VIII) and C (VI-XVIII) must have arisen independently. Thus, at least four independent outcrossing events occurred: C/E (VI-VIII), C (VI-XVIII), R (I-XIX) and V (XIII-XII). Independent hybridizations (an extreme form of outcrossing) are not uncommon in nature, as parthenogenesis triggered by hybridization appeared at least three times in two insects from the Old World, namely the weevil *Otiorhynchus scaber* [4] and leaf beetles of the genus *Calligrapha* [47].

Although parthenogenesis in *N*. *cervinus* is likely to have existed for a long time [20], our study reveals that both males and females would have expanded their range southward down to the mouth of La Plata river, according to what was postulated by [21]. Secondary contact between Forest and Grassland clades would have occurred when favorable climatic conditions returned after allopatric differentiation (Fig 1C), which was subsequently followed by a southward expansion of heterozygous genotypes to Be, BL and LP locations (Fig 1). Considering that the E (VI-VIII) lineage is a derivative from C (VI-VIII) (see Methods section), it is most likely that mating between females C-VIII and males bearing the genotype VI occurred before about 190 kya, a date which roughly coincides with the time span of the secondary contact (100–330 kyr [21]). Therefore, POP individuals may be seen as a testimony of secondary contact in the Paranaense Forest between two clusters that had been isolated from each other for a long time, and allow us to recognize a hybrid zone in this region.

In areas partially overlapping with the one identified in this work, the possible occurrence of a secondary contact between highly divergent clades has been reported for the Rufous-collared sparrow [48] and the little fire ant [49]. In addition, [50] suggested the co-existence of multiple cryptic species among 18 species of butterflies inhabiting this area, similar to what we found in a previous work for lineages I and II of *N*. *cervinus* [28]. Thus, multiple evidence points to this area as a hotspot of instability during Pleistocene-Holocene times.

Recent anthropogenic disturbance has been suggested as a possible explanation for the convergence of hybrid zones in specific geographic locations [51–52], with a tendency to cluster in areas between glacial refugia [52–53]. The term "suture zones" [54] was coined for those geographic areas where numerous long-isolated lineages and closely related species (e.g. cryptic species) made secondary contact and that contain disproportionally high numbers of phylogeographic breaks. Lineages in suture zones diverging due to isolation in Pleistocene refugia were suggested to have concomitant expansion times rather than divergence times, these expansions having been taken place in early to mid-Holocene times [55], as is the case for *N*. *cervinus* [21] and the little fire ant [49]. This assumption provides some additional support for the existence of a suture zone in the area under consideration. The Paranaense Forest, which is one of the most important biodiversity hotspots of the world [56], is now seriously threatened. Our results, together with those reported for other species [48,50], emphasize its role as a valuable system for investigating evolutionary processes, thus making it worthy of conservation.

There are various expected outcomes when two allopatric populations undergo secondary contact, such as complete reproductive isolation [57], reinforcement [58], hybrid speciation [59] and panmixia [48]. However, the results obtained herein do not fit with any of these possibilities. The Fuller’s rose weevil shows a pattern in which heterozygous individuals appear to be fixed heterozygotes, as suggested by the lack of changes in allelic sequences since the proposed time span from outcrossing to present-day. Therefore, heterozygosity has apparently remained "frozen", a phenomenon that can only be explained, to our knowledge, by parthenogenetic reproduction. Then, after the occurrence of the secondary contact, females involved in the outcrossing could have been either bisexual individuals whose offspring subsequently became parthenogenetic, or unisexual individuals who retained the ability to reproduce sexually. Under the first scenario, it is unlikely to recover the same four allele combinations (among more than 400 different possible combinations between Forest and Grassland alleles) from several individuals, because recombination and segregation would have altered this situation through successive generations, while under the second scenario, which implies occasional sex, alleles would have remained unaltered due to the lack of recombination mechanisms [60]. An assessment of ploidy levels in the sample under study would have been helpful to further elucidate the reproductive mode of the maternal lineages involved in outcrossing events. While diploid heterozygotes would have been a strong evidence of outcrossing involving bisexual females, triploid (or higher levels) heterozygotes would have been a hint for outcrossing between unisexual females with unreduced eggs an males producing haploid sperm. Unfortunately, we were not able to analyze ploidy levels in this work because embryos 48 hs old are necessary to quantify this variable and most of the females included in our survey were already dead at the moment of the study.

The stability of a hybrid zone is influenced by factors such as hybrid fitness, rate of dispersal of parental lineages, habitat heterogeneity and environmental change [61]. As previously mentioned, we recovered only four of approximately 400 possible genotypic combinations resulting from interclade mating. The persistence of these four genotypes in nature may be due to a comparatively higher fitness (high heterozygosity resulting from the combination of divergent parental genomes and other particular traits that would have allowed them to out-compete the remaining heterozygous combinations), or to chance. Heterozygotes could also have derived from intraclade crosses, but no POP individuals with both alleles coming from the same clade were ever found (Fig 3). Their absence might be explained by the inability of the sperm to fertilize oocytes of parthenogenetic females, as observed in experimental studies [10, 62–64]. However, our results preclude any outcrossing incompatibility between individuals from different clades.

Although convincing evidence for an infectious parthenogenesis in Naupactini is still pending, the possible role of *Wolbachia* as an inductor of unisexual reproduction in *N*. *cervinus* [26–27] makes it a possible candidate to explain the occurrence of interclade-but not intraclade-mating in this host. Taking into account that vertical transmission of *Wolbachia* allows identifying only the maternal contribution, we conclude that both Forest and Grassland mothers had been infected with the same strain. Thus, the aforementioned mechanism of incompatibility would not be related to the *Wolbachia* strain *per se*, but instead to some other factor intrinsic to the male-female interaction. Further studies are needed to clarify these points.

In brief, our results suggest that two divergent lineages of *N*. *cervinus* would have mated successfully while undergoing secondary contact in the Paranaense Forest, which emerges as a possible suture zone worthy of investigation. High levels of heterozygosity persisted over time in daughter clones through parthenogenetic reproduction. It still remains to be demonstrated the heterozygosity of these individuals across the whole genome. Ongoing genomic studies in *N*. *cervinus* will attempt to test this hypothesis.
